# Primary retroperitoneal mucinous cystadenoma with borderline malignancy in a male patient: a case report

**DOI:** 10.1186/1757-1626-2-9098

**Published:** 2009-11-27

**Authors:** Aicha Benkirane, Asmaa Mikou, Ahmed Jahid, Fouad Zouaidia, Laila Laraqui, Zakia Bernoussi, Najat Mahassini

**Affiliations:** 1 Pathology Departement. Ibn Sina Hopital. Mohamed V University, Rabat, Morocco

## Abstract

**Introduction:**

Primary retroperitoneal mucinous cystadenoma is a rare tumor prevailing specifically in female gender. Its histogenesis is still unclear and its diagnosis is mainly based on morphological characteristics.

**Case presentation:**

the subject is a 44 years old man presenting an abdominal pain on the right side, with a palpable mass which appeared four months ago. Abdominal ultrasound (echography) revealed a retroperitoneal cystic process, which was successfully resected through laparotomy.

Histopathological examination concluded to a mucinous cystadenoma with borderline malignancy foci.

After a year of follow-up, no relapse was noticed in this patient.

**Conclusion:**

Retroperitoneal mucinous cystadenoma is a rare tumor that should be considered in front of a retroperitoneal cystic process. Several hypotheses may explain the histogenesis of this pathological process.

The interest in publishing this case report on primary retroperitoneal mucinous cystadenoma in a male patient lies in the rarity of occurrence of this syndrom in males as compared to females.

## Introduction

Primary retroperitoneal mucinous cystadenoma is a benign and rare tumor. Only 47 cases have been reported in the literature worldwide [1]. Retroperitoneal cystic mucinous tumors have the same macroscopic, morphologic and ultrastructural characteristics than the ovary mucinous processes [2,3]. Retroperitoneal borderline mucinous cystadenoma belongs to the histological spectrum going from benign forms (mucinous cystadenoma) to malignant ones (mucinous cystadenocarcinoma) [2,4,5].

## Case presentation

The patient is a moroccan man of North African origin aged 44, with no significant pathological history. He had never drunk alcohol and had smoked only for a while when he was still at school.

He was presenting a torsion-like pain on the right side of his abdomen over the last four months and had lost weight in the last few weeks.

The clinical examination revealed palpable process, measuring 15 cm (large axis) on the right side. There was no additional noticeable abnormality such as testicular ectopia during the examination.

**Figure 1:**
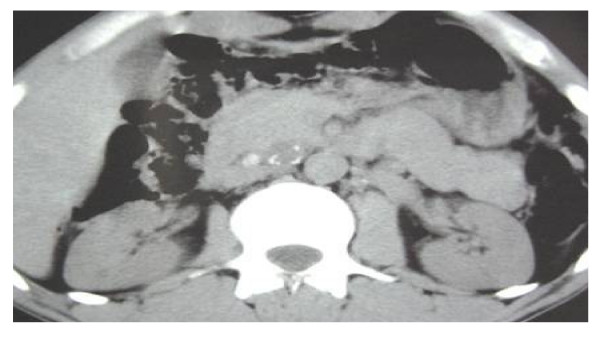
**CT scan demonstrating several retroperitoneal masses, located in pre-aortic and inter aortocave, measuring between 2 and 5 cm, cystic aspect with some heterogenic focuses**.

CT scan and abdominal echography showed several retroperitoneal masses, located in the pre-aortic and inter aortocave areas, and measuring between 2 and 5 cm. They had cystic aspect with some heterogenic focuses. (Figure 1)

Biological analysis (blood formula, ionogram, prothrombin rate) were normal.

A tumoral exeresis has been performed.

The macroscopic analysis of the resection fragment showed 4 portions, the biggest of them measured 5 × 4 × 3 cm in its wide axis. It was polycyclic, with an elastic consistency, and surrounded by an intact, thin capsule.

**Figure 2:**
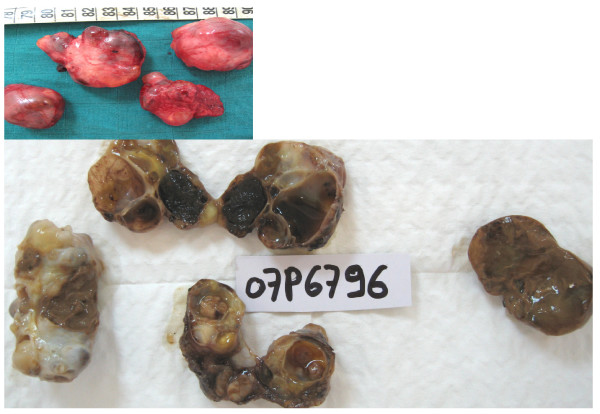
**Macroscopic aspect: 4 portions, the biggest measures 5 × 4 × 3 cm in its large axis, was polycyclic, with elastic consistency, circled by an intact and thin capsule**. At the opening: multilocular cystic aspect with solid zones and endokystic vegetation. The inside of the cysts was gelatinous.

At the opening: a multilocular cystic aspect with solid zones and endokystic vegetation was disclosed. The inside of the cysts was gelatinous. (Figure 2).

Microscopically, the cyst was lined by a single layer of tall columnar epithelium with clear cytoplasm and small nuclei that were basely located in the cells. In another site, the mass was a borderline mucinous tumor similar to that generally observed in the ovary.

**Figure 3:**
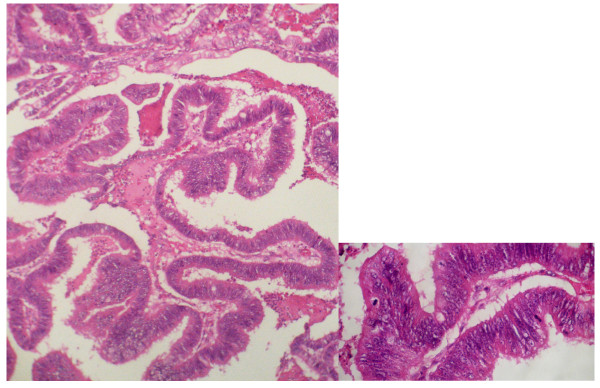
**Single layer of tall columnar epithelium arising stratified epithelium without invasion**. The component cells are mucinous-type with moderate cytological atypia. Hematoxylin and eosin staining; original magnification x100 for the biggest microphotography and x400 for the other.

The cyst was lined by mucinous epithelium with mild to moderate stratification, with mild to moderate cytological atypia; the cystic wall was fairly thin (Figure 3). Although some features of benign mucinous tumor were seen, ovarian tissue was not identified. Immunohistochemically, the lining cells of the cyst were positive for cytokeratin 7 and cytokeratin 20.

The diagnosis was a primary retroperitoneal mucinous cystadenoma with borderline focuses. There is no relapse throughout a twelve months follow-up period.

## Discussion

Retroperitoneal mucinous cystadenoma with borderline focuses is an extremely rare tumor. So far, only 14 cases have been reported in the literature [1], all but one among them were reported in women. Our observation concerns the second male patient affected with this pathological disorder (worldwide). The characteristics of the reported cases are summarized in table 1. Cases include women aged 17 to 48 years and one man who was 63. The mean age is 39 years., All patients were admitted for flank pain with an abdominal distension. Unlike the above cases, the patient subject of the present study, who was 44, presented abdominal pains with a right hypochondric process.

**Table 1:** cases of primary retroperitoneal mucinous cystadenoma of borderline malignancy reported in the literature.

Cases	Age (years)	Sex	Clinical presentation	Site/size (cm)	Histology	Evolution
1[10]	41	F	abdominal pain with distension	12 × 10 × 9 cm	primary retroperitoneal mucinous cystadenoma of borderline malignancy	No relaps. Non precised period

2[3]	45	F	abdominal pain	15 cm	PRMC	No relaps at 12 months follow-up

3[7]	36	F	abdominal distension	Right; Periappendiceal/12 × 8 cm	primary retroperitoneal mucinous cystadenoma of borderline malignancy	No relaps at 6 months follow-up

4[2]	41	F	Right flank pain with abdominal distension	Right; displacing ascending colon/21 × 20 × 16 cm	PRMC	No relaps at 18 months follow-up

5[8]	48	F	Post prandial fullness	Right; Ascending colon mesentery;/15 × 13 × 9 cm	PRMC	No relaps at 12 months follow-up

6[11]	33	F	Left flank mass with abdominal distension	Left; lateral to descending colon; 15 × 13 × 8 cm	PRMC	No relaps at 12 months follow-up

7[5]	33	F	Abdominal swelling, pain	Left flank retroperitoneum;/33 cm	PRMC	No relaps at 10 months follow-up

8[12]	38	F	Left flank pain	right, descendant colon mesentery/11 cm	PRMC	metastases to mediastinal lymph nodes, 4 years after diagnosis

9[12]	47	F	flu-like symptoms	Left; loosely adherent to spleen; 13 cm	PRMC	No relaps

10[13]	63	M	abdominal pain	right, peri-nephric/6 × 4 × 3 cm	PRMC	No relaps

11[4]	22	F	abdominal pain and distension	abdomino-pelvian mass/20 × 17.5 × 15 cm.	PRMC	No relaps

12[1]	35	F	Pelvis pain	Retroperitoneal 24 × 25 cm	PRMC	No relaps at 24 months follow-up.

13[14]	47	F	No precised symptom	No precised site 13 cm	PRMC	No relaps at 18 months follow-up

14[15]	17	F	No precised symptom	No precised site 17 × 14 × 6 cm	PRMC	paraovarian recidive after 21 months of surgery

*Present * *case*	44	M	Mass, right hypochondre pain	Right,4 portions of 5 × 4 × 3 and 4 × 4 × 3 and 3 × 3 × 2 and 2 × 2 × 2 cm	PRMC	No relaps at 12 months follow-up

F: female; M: male. PRMC: primary retroperitoneal mucinous cystadenoma of borderline malignancy

Radiologically, retroperitoneal mucinous cystadenoma presents a cystic formation, uni or bilocular repressing the organs around [6].

The differential diagnosis with retroperitoneal mucinous cystadenoma is made with lymphangioma, kystic teratoma, lymphocela, urinoma, kystic mesothelioma [6]. The tumor diagnosis lays on anatomopathologic exam [2].

Macroscopically, we notice several cysts with different sizes from 6 to 33 cm (mean of 14), surrounded by a thin capsule, with no communication with the neighbouring organs. When opened, the cysts have a gelatinous content, they may be uni or multilocular and contain vegetations.

A correct sampling of the resection piece is necessary in order to search for a borderline or a carcinomatous focus [6].

Microscopically, the tumor resembles ovarian mucinous cystic neoplasms. The cyst locules are lined mainly by a single row of mucin-secreting columnar epithelium. The lining epithelium show mild to moderate stratification (not more than 4 layers thick) with mild to moderate cytologic atypia with no evidence of stromal invasion.

The immunohistochemitry analysis shows a positive match to CK7 and CK20 antibodies. This is the same profile encounted in ovary mucinous tumors [6].

The histogenesis of this tumor is still unkown. Several theories do exist. For some authors [7], retroperitoneal mucinous cystadenoma develops at the expense of ectopic or auxiliary ovary in retroperitoneal position. For others, the origin is a monodermic teratoma with mucinous epithelium proliferation [7].

The latest hypothesis concerns a metaplasic origin of the cysts: they may develop from a coelomic epithelium [7]. During the embryogenesis, coelomic epithelium converts to peritoneal mesothelium and ovarian germinal epithelium. Peritoneal mesothelium seems to keep the same differentiation properties than ovarian epithelial tumors [5]. The similarity of immunohistochemical and ultrastructural profiles with ovarian mucinous tumors supports this hypothesis.

The management of this tumor requires a large surgical exeresis by a complete enucleation [2,8,9], with a long term follow up, especially when borderline foci are found, to prevent relapses or malignant degeneration [4].

The postoperative evolution is generally well [7]. One case on 14 developed metastases to mediastinal lymph nodes, 4 years after surgery, and another presented a paraovarian relapse 21 months after the resection.

## Conclusion

Retroperitoneal mucinous cystadenoma is a rare tumor that should be evocated in front of a retroperitoneal cystic process. Several hypotheses may explain its histogenesis. The complete exeresis is recommended, to avoid relapses or a possible evolution to malignant process. Its diagnosis lays on the anatomopathologic examination, requiring a correct sampling, in order not to miss an associated carcinoma.

## Consent

Written informed consent was obtained from the patient for publication of this case report and accompanying images. A copy of the written consent is available for review by the Editor-in-chief of this journal.

## Competing interests

The authors declare that they have no competing interests.

## Authors' contributions

All authors were involved in patient's care. AB, AM analyzed and interpreted the patient data regarding the pathological findings of the patient and prepared the manuscript. AB, AM and AJ edit and coordinated the manuscript. All authors read and approved the final manuscript.
